# Potential roles of extracellular vesicles in the pathophysiology, diagnosis, and treatment of autoimmune diseases

**DOI:** 10.7150/ijbs.39629

**Published:** 2020-01-01

**Authors:** Jing Tian, Giacomo Casella, Yuan Zhang, Abdolmohamad Rostami, Xing Li

**Affiliations:** 1 National Engineering Laboratory for Resource Development of Endangered Crude Drugs in Northwest China, Key Laboratory of the Ministry of Education for Medicinal Resources and Natural Pharmaceutical Chemistry, College of Life Sciences, Shaanxi Normal University, Xi'an, Shaanxi 710119, China;; 2 Department of Neurology, Thomas Jefferson University, Philadelphia, PA 19107, USA.

**Keywords:** Extracellular vesicles, autoimmune diseases, biomarkers, communication, therapy.

## Abstract

Since extracellular vesicles (EVs) were discovered in 1983 in sheep reticulocytes samples, they have gradually attracted scientific attention and become a topic of great interest in the life sciences field. EVs are small membrane particles, released by virtually every cell that carries a variety of functional molecules. Their main function is to deliver messages to the surrounding area in both physiological and pathological conditions. Initially, they were thought to be either cell debris, signs of cell death, or unspecific structures. However, accumulating evidence support a theory that EVs are a universal mechanism of communication. Thanks to their biological characteristics and functions, EVs are likely to represent a promising strategy for obtaining pathogen information, identifying therapeutic targets and selecting specific biomarkers for a variety of diseases, such as autoimmune diseases.

In this review, we provide a brief overview of recent progress in the study of the biology and functions of EVs. We also discuss their roles in diagnosis and therapy, with particular emphasis on autoimmune diseases.

## Introduction

Extracellular vesicles (EVs) are broadly classified in three sub-classes, depending on their biogenesis and size, exosomes (10-100nm); microvesicles (MVs), also called ectosomes, (100-1000nm); apoptotic bodies (1000-5000 nm) 1. However, even if this nomenclature is extensively used, the classification of these heterogeneous populations of EVs is still a matter of debate 2. Our initial understanding of EVs originated from exosomes found in sheep reticulocytes in 1983 3. EVs represent a direct means of cellular communication 1. They thus play an important role in the development and regulation of many biological processes 4. They carry proteins, DNA and various RNAs: mRNAs, miRNAs, long non-coding RNAs (lncRNAs), circular RNAs (circRNAs), all involved in intracellular signal transduction 5. New research evidence suggests that mitochondria may also be contained in EVs, and that these mitochondria are capable of inducing epigenetic modifications of target cells in target organs, such as mesenchymal stem cells (MSCs) altering the macrophage phenotype by EVs-mediated mitochondrial transfer 6. To induce an effect, EVs must interact with target cells, either directly with the plasma or with the endosomal membrane after cellular uptake 1. Thus far, different mechanisms for EVs uptake have been proposed 7.

Given that, theoretically, every cell in the body releases EVs and that in pathological conditions their amount may increase, they represent an excellent biomarker for several diseases 5. In fact, thanks to their properties, EVs can be detected in many different biological fluids such as blood, saliva, urine, semen, bile, cerebrospinal fluid (CSF), amniotic fluid, ascites and breast milk 8-10. Finally, thanks to their biological characteristics, the idea of using EVs as a therapeutic delivery vehicle in many applications has recently emerged 11. Indeed, the ability to deliver molecules in different biological districts hardly accessible such as central nervous system (CNS), without showing immunogenicity, candidates EVs as future therapy. So far, several approaches using EVs as therapy have been proposed 12-15, and many of them have shown promising results in animals, giving chances to use them in humans 16. At present, there are 477 clinical trials underway using EVs in different pathologies according to https://clinicaltrials.gov. We have summarized a portion of the clinical trials related to EVs in Table 1.

### The biogenesis of Extracellular Vesicles

The biogenesis of EVs is similar but not exactly the same 9. Exosomes are the smallest particles and are mainly composed of ceramide, a lipid produced from sphingosine 1. Exosomes are formed intracellularly via endocytic invagination (early and late endosomes) and are released into a structure known as the multivesicular body (MVB), resulting in the progressive accumulation of intraluminal vesicles (ILVs) 5. The MVB can undergo lysosome acidification and destruction or fusion with the plasma membrane, releasing its cargo of exosomes a mechanism regulated by Rab GTPases proteins 17, 18. Endosomal sorting complexes required for transport (ESCRT) is a mechanism of ILVs or MVB production 19. ESCRT is a protein complex located on the cytoplasmic side of the endosome; its main function is to sort cell components into ILVs, forming the precursor of the exosomes. ESCRT contains four main complexes (ESCRT-0, I, II, III) and accessory proteins (such as vacuolar protein sorting 4, VPS4; vesicle trafficking 1, VTA1; ALG-2 interacting protein X, ALIX), all involved in exosomes biogenesis 20. The ESCRT complex functions in a certain order. First, ESCRT-0 and ESCRT-I limit the ubiquitinated transmembrane cargo subunits to the micro-domains of MVE or ILVs, while causing deformation of the membrane structure, ESCRT-II is then used to recruit ESCRT-III for the budding and scission of this micro-domain 21, 22. The classical ESCRT pathway interacts with syntenin and the ESCRT accessory protein ALIX, and bridges the ESCRT-III subunit VPS32, ultimately performing the scission function of the vesicles to form a closed vesicle 21. The protein of the tetraspanin family is involved in the sorting of vesicle contents 21.

Microvesicles are the EVs class that has been the least studied and thus far we know very little about their biogenesis. They are usually larger than exosomes, depending on the cell type 1. MVs are released by the direct budding or protrusion of the plasma membrane. They typically originate at the plasma membrane in the region called lipid rafts, which are membrane domains rich in cholesterol and glycosphingolipids 23. The formation of MVs primarily involves membrane components and the cytoskeleton 9. Moreover, during MVs formation there is externalization of the phospholipid phosphatidylserine (PS) 24, which normally resides exclusively in the inner layer of the plasma membrane 18.

Currently, no protein markers have been identified for distinguishing exosomes and MVs clearly. However, it has been extensively demonstrated that exosomes fractions are enriched by Alix, tumor susceptibility gene 101 (TSG101), CD81, and syntenin-1 proteins, whereas MVs fractions are enriched by integrins, metalloproteinases, and high levels of PS exposed to the outer membrane leaflet 2, 25. Hence, the following two tables summarize the differences in composition and potential protein markers for different classes of EVs. Table 3 is based on the work of Kowal J et al., which combines gradient centrifugation with iodixanol gradients to separate different sizes of EVs (light sEV, dense sEV, large EVs) and perform western blot (WB) detection 26. Dissimilarly, Yusuke Y et al. performed WB detection on the total EVs obtained by ultracentrifugation of 9 different cells, analyzed the abundance of different kinds of conventional protein markers in the cell lysates and corresponding total EVs 27. The results indicated that CD9 and CD81 are highly enriched in various EVs regardless of the corresponding protein abundance in parent cells. This slightly different result may be due to differences in the methods of extraction and comparison of WB, which also reminds us that various types of EVs need to be standardized protein markers.

As for the release of EVs, a small fraction of EVs will be dissolved in the extracellular fluid with their cargoes, while most of them will interact with specific types of cells. This interaction is not random, but depends on the expression of some specific receptors on the cell surface, although these receptors are not fully identified 28, 29. Moreover, the interaction of most EVs with this specific cell occurs not only near the release site, but also at regions far away from it. Through this interaction, a portion of EVs will bind directly to cell surface receptors, while others will activate receptors to trigger intracellular signaling, some that bind to the receptor and fuse with the target cell membrane, and then release the cargo into the cytoplasm; another portion of EVs will enter the cell by reverse extracellular fusion of the target cell membrane, this fusion occurring on the surface or being internalized by endocytosis or phagocytosis. Once endocytosed or phagocytosed, EVs can be degraded and their components captured by cells for their own physiology. When these cargoes are mixed with local molecules, they participate in the production of other EVs that are similarly released or fused to other cells. This process is defined as a recycling of intercellular communication 28-30.

### EVs isolation and characterization methods

In the last few years, many techniques for EVs isolation and characterization have been described in the literature, many of which, however, have not been well standardized 31. To address this issue, the International Society for Extracellular Vesicles (ISEV) has published a paper on the minimal experimental requirements for definition of EVs and their function (MISEV) 32. We list below the methods most widely used in EVs experimentation.

Isolation: (1) Ultracentrifugation, including differential centrifugations for isolating different EVs fractions, is by far the primary isolation method in EVs application 31. It removes cells and debris under low centrifugal force (300 ×g ~ 2000 ×g) and collects EVs under high centrifugal force (110000 ×g). The disadvantage is that the instrument is expensive, the extraction takes time and effort, and the purity is low. (2) Density gradient is used for isolating different fractions according to their different density. It is a more rigorous method than ultracentrifugation and is usually used to separate exosomes because of its high purity characteristics, but it cannot meet the requirements of large amount of samples 33. (3) Co-precipitation technique relies on the ability of the customized polymer to co-precipitate EVs at lower centrifugation rates 34, 35. This method easily causes co-precipitation of non-EVs components and greatly reduces the purity of EVs. Although commercial kits have been developed, they are still not suitable for large-scale use. (4) Size exclusion-chromatography (SEC) takes advantage of the principle of gel chromatography and the EVs are mainly purified by weight or size, it has a high yield but low specificity 34, 35. Although these methods represent the most suitable ones proposed thus far, there is uncertainty about their application and efficacy in clinic, where the source of EVs samples is very often limited.

The most commonly used method is ultracentrifugation, Ludwig AK et al. combined polyethylene glycol (PEG) co-precipitation and ultracentrifugation to obtain EVs from cell culture supernatants, which can obtain ideal EVs in large quantities without affecting the results of *in vivo* experiments^36^. This method also optimizes the shortcomings of PEG co-precipitation and has reproducibility and scalability, but the final product still contain several non-EV related molecules, which are less pure than size exclusion chromatography and sucrose density gradient centrifugation. At present, microfluidic filtering techniques are considered the most promising strategies for EVs separation in clinic based on their expandable flux, high degree of automation, and solid reproducibility 34, 37. Liu C et al. introduced a method for EV purification by microfluidic filtering combined with droplet enzyme-linked immunosorbent assay (ELISA), which fills the gap in accurate quantification of EVs 38. Wu et al. introduced a method based on the acoustic principle combined with microfluidic technology whereby it would become relatively simple to isolate EVs from whole blood, in a label-free and contact-free manner that would protect the peculiarities, structures and functions of isolated EVs 39. Another novel technique for EVs purification proposed by Kabe et al. 40, consists of a high sensitivity and linearity method from human serum using the properties of magnetic nanobeads and optical disc technology.

Characterization: WB remains by far the common used method for characterizing EVs; also, in this case, the ISEV has published guidelines for the identification of EVs, using antibody for proteins like CD63, CD9, CD81, TSG101, HSP70, Alix 9. For studying EVs size and morphology, reliable methods are electron microscopy (EM) techniques such as transmission electron microscopy (TEM), scanning electron microscopy (SEM), Cryo-electron microscopy (Cryo-EM), and atomic force microscopy (AFM). Confocal fluorescence microscopy (CFM) is another advantageous technique for studying the interaction between EVs and cells 41-43. This technique has resulted very convenient to study the internalization of EVs by recipient cells 42-46. Mrinali et al. developed an innovative method that combines formalin with 1-ethyl-3-(3-dimethyl aminopropyl) carbodiimide (EDC) to improving EV imaging *in situ*. This approach avoids the traditional formalin-based technique, which may reverse cross-linkage due to temperature and allow EVs to escape from the tissue, resulting in a negative or low signal 47. Therefore, this technology has dramatically improved EVs studies related to basic science, and perhaps, it will be used for clinical studies. Furthermore, Nanoparticle Tracking Analysis (NTA) and Tunable Resistive Pulse Sensing (TRPS) are common techniques for EVs quantification and size distribution 48. NTA is able to track the scattering of individual vesicles over time, while TRPS is the change in the instantaneous current when the vesicles pass through the pores 49, 50. According to the work of Akers JC et al, exosomes are more suitable for NTA, and MVs is more suitable for TRPS 51. Flow Cytometry is also a widely use method for characterization and quantification 52, especially now that there is a new fluorescence activated cell sorting machine with a detection limit around 100 nm (Cytoflex) 34.

### EVs as therapy

Our current knowledge on EVs provides opportunities for their use in therapy. The relevance of EVs as a therapeutic tool, already established in the last few years, is still growing and promising important developments for the near future. Many approaches have already been developed 5, 12, 35, 53-55. EVs act an important role in the therapeutic effect of MSCs and are therefore considered as a potential alternative to MSCs. Thus, MSC-derived EVs (MSC-EVs) would induce a regulatory response in the function of T-, B-, and monocyte-derived dendritic cells 56. In the present work, MSC-EVs have been shown to possess immunomodulatory functions which promote B cell activation, induction of Breg and B cell proliferation was compared to that the whole MSCs 57. Fujii et al., showed EVs derived from BM-NSCs have potential therapeutic effect on acute graft versus host disease (GVHD) and indicated the EVs probably inhibit the effector T cell induction and kept circulating naïve T cells 58. However, EV usually does not treat as well as the cell itself, and its biological effects may be affected by the surrounding microenvironment 59. In this case, Milad et al., found exosomes derived from MSCs stimulated by IFN-γ displayed a superior therapeutic effect compared with native exosomes. This study indicated that MSCs activated by IFN-γ, which promote IFNγ-Exo loaded more immunosuppressive cytokine indoleamine 2,3 -dioxygenase (IDO) resulted in peripheral blood mononuclear cells (PBMCs) proliferation disability. Therefore, this “pre-stimulation” of EVs greatly enhances its therapeutic effect and represents a new approach for treatment.

The most intriguing aspect of EVs to have attracted the scientific interest is the possibility of customizing them “ad hoc” 13. This means loading EVs with different functional molecules simultaneously 60, targeting EVs membrane for increasing tissue and cell targets 61. We list here the most recent and promising studies published so far. Kamerkar et al. engineered exosomes (named in the paper iExosomes) to specifically target pancreatic cancer cell-associated KRAS mutant genes, delivering RNAi and CD47 on exosomes 55. Directly specific targeting of KRAS was not possible to achieve before this study. On the other hand, Kumar et al. demonstrated an approach whereby EVs are encapsulated by a 10 nm thick protective film formed by a supramolecular complex of ferric ions and tannic acid to achieve non-essential EVs loss, and the protective film can be fused with other molecules as targeted delivery while the protective film is also subject to controlled degradation 62. Longatti et al. published a method for specifically targeting exosomes membrane, with a single-chain variable fragment of antibody 63. Recently, Jia G et al. demonstrated a method for treating glioma cells, using a click chemistry method to engineer exosomes with neuropilin-1 peptide and load them with curcumin 60. Similarly, Tian et al. used a strategy for treating murine ischemia targeting exosomes with c(RGDyK) peptide 13.

However, there are still two problems that need to be solved: the methods for loading drugs into EVs cannot guarantee high loading efficiency 64, and, as a therapeutic medium, EVs cannot be targeted to the lesion area very well for therapeutic effects 65. Thus, improving the EVs loading and delivering efficiency is one aspect that has been reconsidered, due to a discrepancy in the published protocol and data reproducibility 66. Piffoux et al. published a method for loading EVs using liposomes and showed impressive results 67. However, the efficacy of this protocol has not been confirmed.

In the diagnosis and treatment of graft-versus-host disease (GVHD), the application of EVs has made more advancement. The EVs isolated from patient serum contains three miRNAs (miR-423, miR-199, miR-93), which may be related to the incidence and severity of GVHD 68; and the expression of CD146, CD31, and CD140-α on EVs surface, is also closely related to the onset of the disease 69. Traditional MSCs therapy after replacement with EVs for GVHD also showed ideal results, suggesting that MSCs-derived EVs are potential for cell-free therapy for GVHD 58, 70. Kordelas L et al. used MSCs-derived EVs instead of MSCs in the patient with GVHD, and the promising results were obtained. MSCs-derived EVs provide a potentially viable method for refractory GVHD 71.

In other diseases, such as cancer, EVs also bring new opportunities for treatment, prevention or diagnosis. Some studies have found that proteins or nucleic acid components in EVs can inhibit tumor development 72, which may be a direct influence or through antigen presentation to immune cells. For example, DCs loaded with exosomes and alpha-galactosylceramide can significantly improve the tumor microenvironment of rats bearing gliomas and increase the median survival of rats 73. In contrast, hepatocellular carcinoma-derived exosomes miR-21 are thought to convert normal hepatic stellate cells to cancer-associated fibroblasts, thereby promoting tumor progression, which may provide new targets for the prevention and treatment of liver cancer 74. The latest evidence suggests that the ability of EVs to tissue regeneration is equally astonishing. Human perivascular stem cell-derived EVs can mediate bone repair 75, and endothelial cell-derived exosomes can induce angiogenesis in ischemic myocardium 75, MSCs-derived EVs are important mediators of cartilage repair, which will have great application prospects as a therapeutic drug for cartilage regeneration and osteoarthritis 76.

### Extracellular vesicles as biomarkers of autoimmune disease

In the CNS, EVs have been observed in the cerebrospinal fluid, indicating their involvement in many autoimmune diseases, such as multiple sclerosis (MS) 77 and neuromyelitis optica 78. They may play important roles in several biological processes such as cell-to-cell communication, immune system response, and nerve degeneration 79-81. In MS, Verderio et al. showed that the number of myeloid EVs was enhanced. These myeloid-derived EVs were considered as diseases marker which separation from plasma and can be used to monitor disease progress 77, 82. Similar works were also proved from plasma and serum and indicated EVs could provide useful information for disease diagnosis. However, serum has been questioned as a biomarker recently. The main comment is that PS-positive MVs in serum are consumed during the promotion of coagulation, and the remaining MVs do not reflect overall MVs levels; platelets also release MVs after activation in coagulation; what's more, the presence of thrombin will also influence MVs surface proteins 83. In summary, plasma rather than serum can lead to more convincing results 84.

Urine is another biological fluid commonly used for new biomarkers assess due to the noninvasive collection approach. Recently, the composition of exosomal miRNA was determined by deep sequencing and several stable expression miRNA were confirmed as biomarkers, which has verified the notion of urinary exosomes as a stable source of miRNA biomarkers for renal fibrosis 85. Thus, EVs may be a novel biomarker that will soon replace biopsies.

There is also evidence that miRNA production and transport are associated with autoimmune diseases, especially MS 86. EVs contain a large amount of miRNA, which is the main carrier of circulating miRNAs 86, and the EVs content in patients' blood is also different from that of healthy controls 87. This thus demonstrates that miRNAs in EVs are a potential biomarker for the diagnosis of MS. At the same time, it also sets forth requirements for the standardization of miRNA collection methods. Recently, the results of sequencing the total circulating exosomes transcriptome in patients with relapsing-remitting MS compared with healthy controls also confirmed that exosomal miRNA is very helpful as a biomarker for patients with MS 88. In the table below, we summarize some of the recent studies on EVs as biomarkers in autoimmune diseases.

### EVs play an important role in autoantigen presentation and T cell modulation

**Figure 1:**
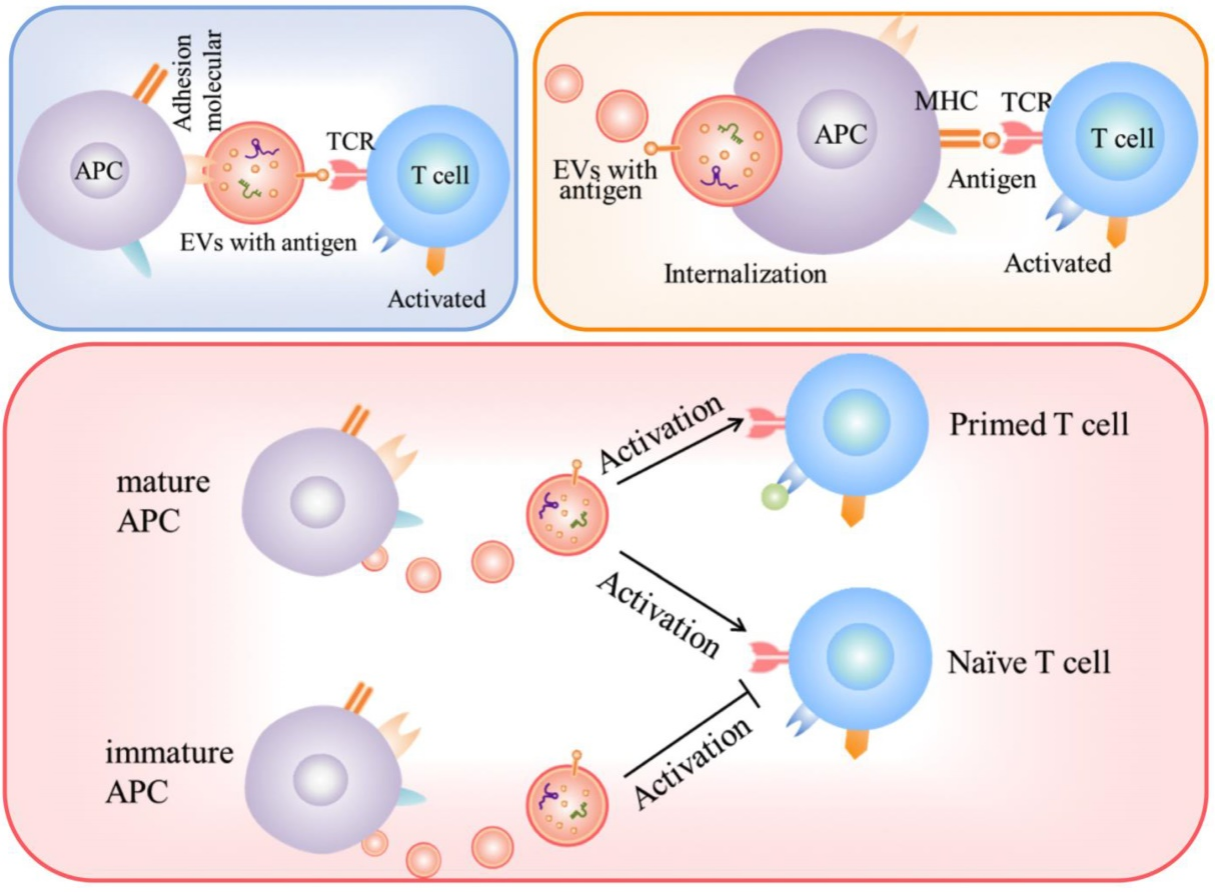
Roles of EVs in autoantigen presentation response.

Given that EVs express peptide-MHC complexes, they may present antigens and activate immune cell-response 16. EVs, in particular, exosomes, have been investigated in antigen presentation. Exosomes released by antigen-presenting cells (APC) may express class I and II MHC molecules and co-stimulation molecules and thus, theoretically, activate CD8^+^ and CD4^+^ T lymphocytes 89. There are three major mechanisms for EVs to participate in autoantigen presentation. EVs participate in autoantigen presentation process involved in autoimmunity is shown in Fig. 1.

Firstly, exosomes might act indirectly from APCs uptaking/stimulation 90. This mechanism appears more relevant for naïve T cell activation. Eventually, costimulatory molecules expressed by APCs provide a necessary second signal for activating T lymphocytes.

Secondly, the exosomes can be internalized by APCs and then present antigen to the surface to form a MHC complex 91, 92. Once internalized by the APCs, the complex peptide/MHC will be exposed to the membrane. Finally, this complex will be involved in T lymphocytes activation.

Thirdly, the study of the immunostimulatory capacity of APC-derived EVs is still a matter of debate. Some groups have shown that EVs can directly activate T cells 93, while others have shown that EV-dependent T cells stimulation is not enough and still requires APCs participation 94.

There are few studies on EVs release in T cell immune regulation mechanisms, but some reports have demonstrated its importance. It is noteworthy that regulatory T cells (Treg)-derived EVs could promote other T cells polarized to the Treg phenotype 95. In addition, Treg effectively exocytosed immunosuppressive EVs that suppressed IFN-γ production and the proliferation of Th1 cells 96. Similarly, EVs from endothelial cells possess the modulation ability which blocks T cells activation and alleviate chronic inflammation in tissue 97. In this case, via the transfer of anti-inflammatory miRNAs, MSCs release immunosuppressive exosomes, which are actually used to treat autoimmunity disease in clinical 98, 99.

### The application of extracellular vesicles in autoimmune diseases

#### Multiple Sclerosis (MS)

MS and its animal model experimental autoimmune encephalomyelitis (EAE) is the most common inflammatory demyelinating disease caused by autoimmune-activated immune cells in the CNS 100, 101. It has been reported that EVs can penetrate the blood-brain barrier (BBB) and contribute to brain antigens spreading to the periphery 102. And the injection of microglia-derived EVs into the CNS of EAE mice enhanced inflammation and exaggerated disease 77. Moreover, mice with an impaired ability to secrete EVs were resistant to EAE 77. Therefore, these data indicate that EVs are involved in EAE mechanisms. Based on the EVs roles in EAE and MS, some groups developed treatment strategies that employed the function of exosomes. Exosomes (IFNγ-Exo) produced by IFNγ-stimulated MSCs have shown a good therapeutic effect on EAE 103. These data provided evidence that MSCs-derived exosomes can be used as cell-free therapies for autoimmune and central nervous system diseases. The use of EVs derived from overexpression of TGF-β1 dendritic cells to EAE showed that Th1 and Th17 differentiation was inhibited and promoted Treg production, which led to diminished expression of EAE 104. Casella G. et al. designed a mouse microglial cell line that releases a large amount of engineered EVs containing anti-inflammatory cytokine IL-4, after injection, the clinical score of EAE is significantly reduced 15. Also, Zhuang et al. delivered curcumin-loaded exosomes through a nasal route to mice with lipopolysaccharide (LPS)-induced encephalitis and reduced neuroinflammation by targeting microglia 14. In other similar LPS-induced inflammatory demyelinating models, curcumin is also encapsulated by exosomes, playing a role in enhancing anti-inflammatory activity 33. In short, regardless of small-molecule chemical drugs, nucleotide drugs or protein drugs, as a drug delivery system, EVs have shown great potential for application in modern medicine. Furthermore, Pusic A. D. et al. implied exosomes released from bone marrow-derived dendritic cells as a therapeutic medium that supported the maturation of oligodendrocytes 105. Dendritic cell-derived exosomes stimulated by low levels of pro-inflammatory factors IFN-γ contain higher levels of miR-219, which is vital for oligodendrocyte precursor cells (OPCs) differentiation; meanwhile, these exosomes are proved increase mature oligodendrocytes and recovery the destroyed myelin *in vivo*. Thus, exosomes might not only exacerbate inflammation during EAE but may also induce myelin regeneration.

#### Rheumatoid Arthritis (RA)

RA is an autoimmune disease in which the immune system reacts against the body's cells and tissues. Generally, synoviocytes-derived exosomes, in inflammatory conditions like RA, stimulate surrounding cells to secrete inflammatory mediators for damaging cartilage 106. It has been shown that the use of exosomes derived from IL-10 treated-dendritic cells, induced amelioration of RA severity 54. IL-1β-stimulated fibroblast-like synoviocytes-derived EVs promoted osteoarthritic changes in chondrocytes 107. In RA models, EVs have exhibited immunological abilities to induce apoptosis, presenting antigen to T cells, and causing extracellular damage 108. All these pieces of evidence have contributed to propose synovium-derived EVs as possible biomarkers for RA 109, 110; indeed, they might predict disease stage and, potentially, become relevant for building new and more effective therapeutic approaches.

The pathogenesis of RA may be related to the communication of EVs cell-to-cell, which involves many complex processes such as antigen presentation and formation of immune complexes 111, inflammation 112, 113, destruction of extracellular matrix 114, delivery of miRNAs 115, 116. Withrow J et al. and Fu H et al. reviewed in detail the research progress of EVs in these aspects 117, 118. It can be concluded that EV mediates crosstalk between immune cells, synoviocytes, endothelial cells, and chondrocytes, thus affecting various processes of RA. These efforts may reveal the pathogenesis of RA, provide new insights into the targeted treatment of RA, and new treatment opportunities, as well as treatment strategies, may be discovered.

#### Type 1 diabetes (T1D)

T1D, also called diabetes mellitus type 1, is a form of diabetes mellitus in which pancreas fails to produce insulin. T1D is primarily a childhood associated autoimmune disease characterized by the destruction of insulin-producing β cells in the pancreatic islets of Langerhans 119. Recent work showed that EVs may be involved in the pathogenesis of T1D playing a role in the presentation of autoantigen peptides from insulin-producing β cells. For example, T lymphocytes release exosomes containing specific microRNAs (for example, miR-142-3p, miR-142-5p, and miR-155) that transfer microRNAs to rodent and human pancreatic β cells, these microRNAs triggers the expression of chemokines in pancreatic β cells to promote apoptosis and result in insulin secretion disorders 120. Exosomes also have shown potential as a therapeutic agent in treating T1D. In particular, exosomes from human urine-derived stem cells can prevent kidney injury in T1D rats by the transfer of growth factors, transforming growth factor-β1, angiogenin and bone morphogenetic protein-7 121. Interestingly, studies of MSCs have consistently shown that MSCs can inhibit autoimmunity in T1D 122, 123. More detailed research data later indicated the positive effect of EVs on these two autoimmune diseases 124. MSC-derived EVs not only inhibits Th1 and Th17 cells but increases the expression of immunosuppressive cytokine IL-10, thereby effectively preventing T1D disease development 124. To further characterize of exosomes, their underlying mechanisms and relationship with different stages of the disease will furnish the understanding of the role of exosomes in the pathogenesis of T1D and their possible application as therapeutic tools.

#### Antiphospholipid syndrome (APS)

APS is an autoimmune disease that can cause venous or arterial thromboembolism or severe pregnancy mortality 125. In the last two decades, researchers have believed that the APS pathogenesis may be related to persistent antiphospholipid antibodies (aPL) 126, and the main difficulty in elucidating the pathogenesis of APS is the heterogeneity of aPL 127. There is strong evidence that EVs in patients with APS are indeed elevated. It is speculated that elevated EVs levels reflect the general state of vascular activation. It has been reported that EVs may stimulate thrombosis and promote vascular activation 128. Analysis conducted in sera of patients affected by APS reported a significant increase in endothelial cell-derived microvesicles 129. Limited experimental data indicate that aPL stimulation can induce endothelial cells to release specific EVs, unlike normal types, which contain IL-1β and specific miRNA molecules. Thus, with the spread of such EVs, the unstimulated endothelial cell was activated by an autocrine or paracrine manner 130. In the APS condition, EVs have shown to induce the development of thrombosis through several mechanisms. For example, microvesicles express a high level of phosphatidylserine 131, and this might facilitate the assembly of calcium-dependent coagulation complexes and supports thrombin generation. Further, EVs are, usually, enriched of tissue factors and may directly support procoagulant activity 132. Recent evidence demonstrated that endothelial microvesicles can interact with blood monocytes and stimulate procoagulant activity 133.

### Systemic lupus erythematosus (SLE)

SLE is a chronic systemic autoimmune disease that influences various organs and systems. SLE is featured by elevated levels of pathogenic autoantibodies, resulting in deposition of immune complexes that cause damage to multiple systems and organs 134, 135. Several studies reported the defective of apoptosis activity in SLE, leading to the development of autoimmunity 136. Moreover, SLE patients exhibit an increased level of IgG-MPs in plasma compared to controls and it correlates to dsDNA antibodies 137. Recent evidence suggests that apoptosis-derived membrane vesicles from the serum of SLE patients may activate cyclic guanosine monophosphate (GMP)-AMP synthase (cGAS) and stimulator of interferon genes (STING) pathway to induce the production of IFN-I 138. It is suggested that blocking the cGAS-STING pathway or inhibiting the secretion of apoptosis-derived membrane vesicles may be a promising therapeutic target for SLE. On the other hand, MSCs infusions technology has been used to treat a variety of diseases. Liu's team showed that MSCs infusions can improve bone loss in Fas-deficient MRL/lpr mice and restore the function of bone marrow-derived MSCs, the mechanism is that MSCs infusions can down-regulate the intracellular level of miR-29b by reusing Fas from MSCs infusions-derived exosomes, thereby restoring the hypomethylation of the Notch1 promoter in SLE patients, suggesting that exosomes may have clinical therapeutic value in saving SLE secondary osteoporosis 139.

#### Hashimoto thyroiditis (HT)

HT is a common autoimmune disease with an annual incidence rate of about 1 per 1,000 people, mainly affecting women 140. The pathogenesis is complex and unknown. Etiology divides HT into primary and secondary forms 141. The pathological features of HT are mainly infiltration of hematopoietic monocytes (mainly lymphocytes), destroying thyroid cells, causing thyroid enlargement, fibrosis or other variants 142-144. Then clinical manifestations of thyroid function decline or even loss 145, causing abnormalities in the gastrointestinal system, cardiovascular system, lung system, hematopoietic system, and nervous system 141. The current research status still cannot explain the pathogenesis of HT, and a recent work discussed whether circulating exosomes participate in the inflammatory response of HT. By detecting exosomes in the serum of patients, it is indicated that these exosomes present antigen to activate DCs, after activation, it leads to an imbalance of CD4+ T cell differentiation, may induce HT, or will become a breakthrough in the pathogenesis of HT 146.

**Figure 2:**
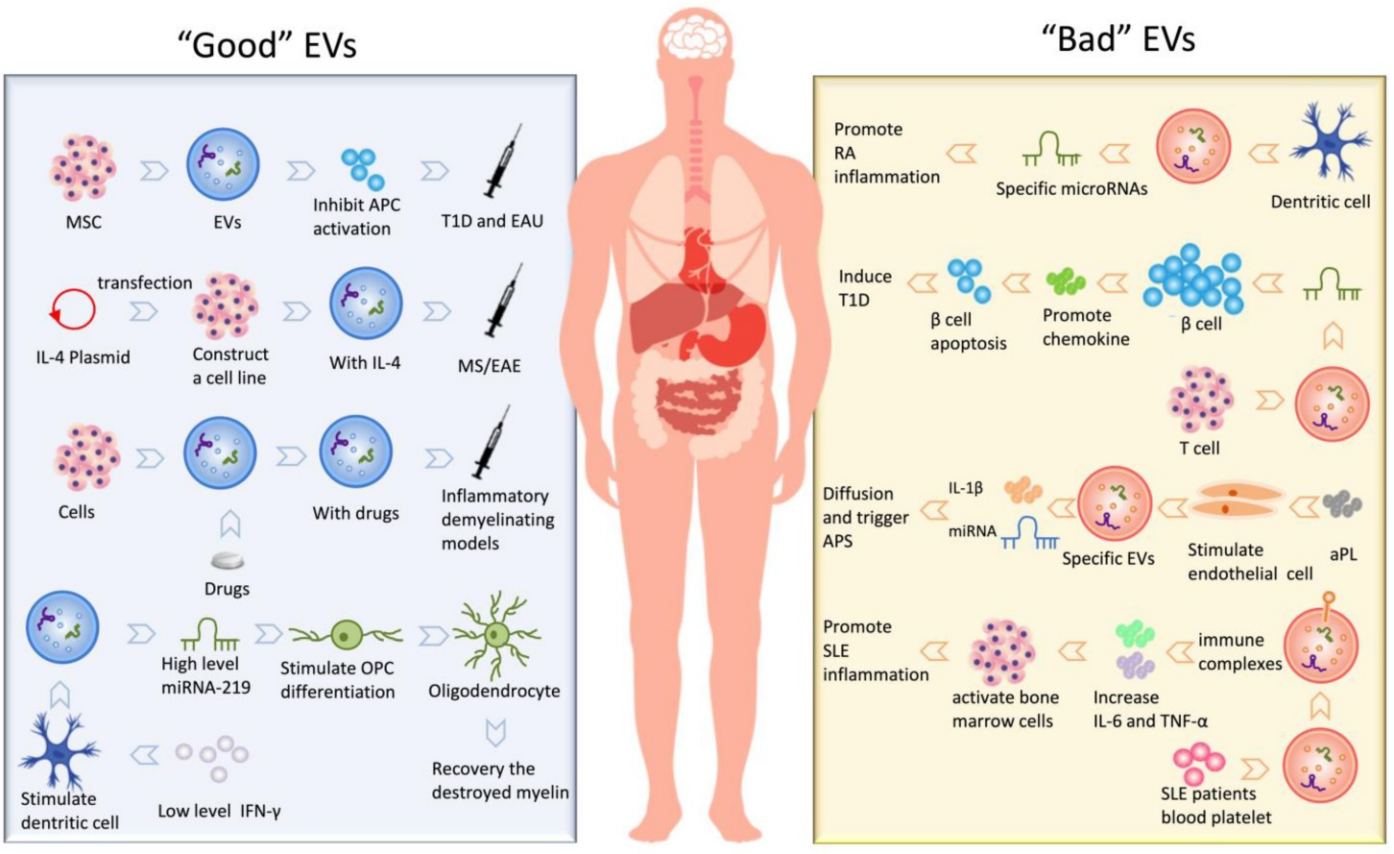
The application of extracellular vesicles in autoimmune diseases.

In short, the existence of EVs is not only involved in the development of many autoimmune diseases but also participated in the treatment of these diseases as a therapeutic intermediary. We have summarized kinds of “Good” EVs that can participate in the treatment and “Bad” EVs that promote the development of the disease, shown in Fig. 2.

## Conclusion

Research on EVs has been ardent since 2007 and has displayed unique advantages in various fields. EVs possess subcellular structures, which load and may deliver a variety of molecules that participate in many physiological processes. These pleiotropic effects may represent a greater breakthrough in the field of autoimmune diseases. However, the current problem to be solved is the standardization of the extraction and purification methods of EVs and the more accurate and effective quantification of the quantity or concentration of EVs. The characterization of EVs has been plaguing researchers, and the existing approaches are more or less flawed. With emerging technologies that can better solve this problem, spending more efforts to determine their origin characteristics, and the pathogenicity role of EVs may pave the way for new diagnostic methods. Given the various advantages of EVs and the current research results, we hope that EVs can also play a paramount role in autoimmune diseases, for example, as a carrier of therapeutic drugs or as a means of diagnosing diseases. This review retrospects the development, characterization, and biogenic derivation of EVs, as well as recent research achievements on autoimmune diseases. It is intended to provide basic ideas and information for scholars who have entered the field.

## Figures and Tables

**Table 1:** Clinical trials related to EVs.

Condition or disease	Origen of EVs	Type of EVs	Phase	NCT number
Type1 Diabetes Mellitus Type2 Diabetes Islet Cell Transplantation	Blood	EVs	ND	NCT03106246
Smoker	Human broncho alveolar lavages	EVs	ND	NCT03608293
Blood Coagulation Disorders	Bone marrow	EVs	ND	NCT00086476
Bronchopulmonary Dysplasia	Bone marrow mesenchymal stem cell	EVs	Phase 1	NCT03857841
Coronary Artery Disease Ischaemic Heart Disease Angina Pectoris	Blood	EVs	ND	NCT03674255
Cancer	Blood	EVs	ND	NCT03262311
Cerebrovascular Disorders	Mesenchymal stem cell	Exosomes	Phase 2	NCT03384433
Ulcer	Plasma	Exosomes	Phase 1	NCT02565264
Pancreatic Cancer	Venous blood	Exosomes	ND	NCT03821909
Colon Cancer	Plant	Exosomes	Phase 1	NCT01294072
Sarcoma	Blood	Exosomes	ND	NCT03800121
Lymphoma	Blood	Exosomes	ND	NCT03985696
Cirrhosis	Hepatocyte	MVs	ND	NCT03837444

ND: Not Determined.

**Table 2:** Composition of exosomes and microvesicles.

Composition	Exosomes	Microvesicles
Proteins	MVB formation: ALIX, TSG101; Tetraspanins: CD9, CD63, CD81,CD82; Membrane transport and fusion: annexins, flotillins, GTPases; Adhesion: integrins; Antigen presentation: MHC class molecules; Heat shock proteins: HSC70, HSP90; Adaptor protein: Syntenin-1	Matrix metalloproteinases; Tetraspanins: CD9, CD63; Glycoproteins: GPIb, GPIIb-IIIa, P-selectin; Integrins: Mac-1; Receptors: EGFRvIII; Cytoskeletal components: β-actin and α-actinin-4; Antigen presentation: MHC class molecules; Heat shock proteins: HSC70, HSP90
Lipids	Ceramide; Cholesterol; Phosphatidylserine; Sphingolipids	Phosphatidylcholine; Sphingomyelin; Phosphatidylethanolamine; Lysophospholipids
Nucleic acids	mRNA; miRNA; Non-coding RNA; DNA	mRNA; miRNA; Non-coding RNA; DNA

**Table 3:** The potential protein markers of exosomes and microvesicles.

	Exosomes	Microvesicles
Markers	ALIX; TSG101; CD81; Syntenin-1; ADAM 10; EHD-4; Annexin XI	Actinin-4; GP96; Mitofilin
Shared markers	Hsc70; Hsp90; Flotillin-1; Annexin II; MHC I; MHC II; CD9; CD63; Actin; Ezrin; Moesin	

**Table 4:** EVs as a biomarker in autoimmune disease.

EVs Origin	Classification	Components	Change	Disease	Reference
Serum	Exosomes	Circulating exosomes	↑	Systemic lupus erythematosus	147
Urine	Exosomes	miR-146a	↑	Systemic lupus erythematosus	148
Urine	Exosomes	miR-29c	↓	Lupus nephritis	85
Serum	Exosomes	Total exosomes	↑	Non-obese diabetes	149
CSF	Microvesicles	Total microvesicles	↑	Multiple sclerosis	77
Serum	Exosomes	miRNA	↓	Multiple sclerosis	88
Serum	Exosomes	Hotair	↑	Rheumatoid arthritis	150
Synovial fluid	EVs	miR-155	↑	Rheumatoid arthritis	115
Saliva	Exosomes	miRNA	/	Sjögren's syndrome	151
Eosinophils	Exosomes	Total exosomes	↑	Asthma	152
CFS	Exosomes	Total exosomes	↑	Multiple sclerosis	153
Platelets, total leukocytes or monocytes	Microparticles	Total microparticles	↑	Multiple sclerosis	87
Serum	Exosomes	Myelin protein	↑	Multiple sclerosis	154
Urine	Microparticles	Total microparticles	↑	Type 1 Diabetes	155

autoantigen presentation response.
